# Conjectures of Sun About Sums of Polygonal Numbers

**DOI:** 10.1007/s44007-022-00030-1

**Published:** 2022-11-02

**Authors:** Kathrin Bringmann, Ben Kane

**Affiliations:** 1 Department of Mathematics and Computer Science, Division of Mathematics, University of Cologne, Weyertal 86-90, 50931 Cologne, Germany; 2 Department of Mathematics, University of Hong Kong, Pokfulam, Hong Kong

**Keywords:** Polygonal numbers, Theta functions, Modular forms, Quadratic forms

## Abstract

In this paper, we consider representations of positive integers as sums of generalized *m*-gonal numbers, which extend the formula for the number of dots needed to make up a regular *m*-gon. We mainly restrict to the case where the sums contain at most four distinct generalized *m*-gonal numbers, with the second *m*-gonal number repeated twice, the third repeated four times, and the last is repeated eight times. For a number of small choices of *m*, Sun conjectured that every positive integer may be written in this form. By obtaining explicit quantitative bounds for Fourier coefficients related to theta functions which encode the number of such representations, we verify that Sun’s conjecture is true for sufficiently large positive integers. Since there are only finitely many choices of *m* appearing in Sun’s conjecture, this reduces Sun’s conjecture to a verification of finitely many cases. Moreover, the bound beyond which we prove that Sun’s conjecture holds is explicit.

## Introduction and Statement of Results

For $m\in \mathbb {N}_{\ge 3}$ and $\ell \in \mathbb {Z}$, let $p_m(\ell )$ be the $\ell$*-th (generalized)* *m*-*gonal number*

$$\begin{aligned} p_m(\ell ):=\frac{1}{2} (m-2)\ell ^2-\frac{1}{2} (m-4)\ell . \end{aligned}$$

For $\ell \in \mathbb {N}$, these count the number of dots contained in a regular polygon with *m* sides having $\ell$ dots on each side. For example, the special case $m=3$ corresponds to triangular numbers, $m=4$ gives squares, and $m=5$ corresponds to pentagonal numbers. There are several conjectures related to sums of polygonal numbers. Specifically, for ${\varvec{\alpha }}\in \mathbb {N}^d$,^1^ we are interested in the solvability of the Diophantine equation

1.1 $$\begin{aligned} \sum _{1 \le j \le d} \alpha _j p_m(\ell _j)=n \end{aligned}$$

with $\ell _j\in \mathbb {N}_0$ or $\ell _j\in \mathbb {Z}$. We call such a sum *universal* if it is solvable for every $n\in \mathbb {N}$. Fermat stated (his claimed proof was not found in his writings) that every positive integer is the sum of three triangular number, four squares, five pentagonal numbers, and in general at most *m* *m*-gonal numbers. In other words, he claimed that the sum $\sum _{1\le j\le m} p_m(\ell _j)$ is universal. His claim for squares ($m=4$) was proven by Lagrange in 1770, the claim for triangular numbers ($m=3$) was shown by Gauss in 1796, and the full conjecture was proven by Cauchy in 1813. Going in another direction, Ramanujan fixed $m=4$ and conjectured a full list of choices of ${\varvec{\alpha }}\in \mathbb {N}^4$ for which the resulting sum is universal; this conjecture was later proven by Dickson [11]. Following this, the classification of universal quadratic forms was a central area of study throughout the twentieth century, culminating in the Conway–Schneeberger 15-theorem [3, 8] and the 290-theorem [4], which state that arbitrary quadratic forms whose cross terms are even (resp. are allowed to be odd) are universal if and only if they represent every integer up to 15 (resp. 290). Theorems of this type are now known as *finiteness theorems*. Namely, given an infinite set $S\subseteq \mathbb {N}$, one determines a finite subset $S_0$ of *S* such that a solution to (1.1) exists for every $n\in S$ if and only if it exists for every $n\in S_0$. Taking $S=\mathbb {N}$, one obtains a condition for universality of a given sum of polygonal numbers. For example, choosing $m=3$ or $m=6$, (1.1) is solvable with ${\varvec{\ell }}\in \mathbb {Z}^d$ for all $n\in \mathbb {N}$ if and only if it is solvable for every $n\le 8$ [6], for $m=5$ it is solvable with ${\varvec{\ell }}\in \mathbb {Z}^d$ for all $n\in \mathbb {N}$ if and only if it is solvable for every $n\le 109$ [13], while it is solvable with ${\varvec{\ell }}\in \mathbb {N}_0^d$ for all $n\in \mathbb {N}$ if and only if it is solvable for every $n\le 63$ [14] and for $m=8$ it is solvable for ${\varvec{\ell }}\in \mathbb {Z}^d$ for all $n\in \mathbb {N}$ if and only if it is solvable for every $n\le 60$ [15].

Here, we consider the question of universality in the case ${\varvec{\alpha }}=(1,2,4,8)$ as one varies *m*. Specifically, we have the following conjecture of Sun (see [21, Conjecture 5.4]).

### Conjecture 1.1

For $m\in \{7,9,10,11,12,13,14\}$ and ${\varvec{\ell }}\in \mathbb {Z}^4$, the equation

1.2 $$\begin{aligned} p_m(\ell _1)+ 2p_m(\ell _2)+4p_m(\ell _3)+ 8p_m(\ell _4) = n \end{aligned}$$

is solvable for every $n\in \mathbb {N}$.

### Remark

A proof of Conjecture 1.1 would give a classification of those *m* for which the sum (1.1) is universal in the case ${\varvec{\alpha }}=(1,2,4,8)$. By direct computation, one sees that $p_m(\ell )\in \{0,1\}$ or $p_m(\ell )\ge m-3$. Using this, one obtains that (1.2) is not solvable for $n=16$ for every $m\ge 20$. For $m=16$, $m=17$, $m=18$, and $m=19$, one finds directly that there is no solution for $n=29$, $n=30$, $n=16$, and $n=17$, respectively. Moreover, for $m\in \{3,6\}$, it is known by work of Liouville [18] that the sum is universal, for $m=4$, it was conjectured by Ramanujan and proven by Dickson [11] that the sum is universal, while for $m=5$ (resp. $m=8$), it was shown by Sun in [20] (resp. [21]) to be universal.

In this paper, we prove that Conjecture 1.1 is true for *n* sufficiently large.

### Theorem 1.2

For $m\in \{7,9,10,11,12,13,14\}$, there exists an explicit constant $C_m$ (defined in Table 2) such that (1.2) is solvable with ${\varvec{\ell }}\in \mathbb {Z}^4$ for every $n\in \mathbb {N}_{\ge C_m}$, with the restriction that $n\not \equiv 4\ \, \left( {\text {mod}} \, 16 \right)$ if $m=12$.

### Remark

To prove Conjecture 1.1 for $m=12$, it suffices to show that (1.2) holds for all $n\in \mathbb {N}$ with $n\not \equiv 4\ \, \left( {\text {mod}} \, 16 \right)$ (see Lemma 5.1). Hence, the restriction in Theorem 1.2 is natural.

By completing the square, one easily sees that representations of integers as sums of polygonal numbers are closely related to sums of squares with congruence conditions. In particular, setting

$$\begin{aligned} r_{m,{\varvec{\alpha }}}(n):&=\#\left\{ {\varvec{\ell }}\in \mathbb {Z}^4: \sum _{1\le j\le 4} \alpha _j p_{m}(\ell _j)=n\right\} ,\\ s_{r,M,{\varvec{\alpha }}}(n):&=\#\left\{ {\varvec{x}}\in \mathbb {Z}^4: \sum _{1\le j\le 4} \alpha _j x_j^2=n,\ x_j\equiv r\ \, \left( {\text {mod}} \, M \right) \right\} , \end{aligned}$$

we have

1.3 $$\begin{aligned} r_{m,(1,2,4,8)}(n)= s_{m,2(m-2),(1,2,4,8)}\left( 8(m-2)n + 15(m-4)^2\right) . \end{aligned}$$

Hence, since Conjecture 1.1 is equivalent to proving that $r_{m,(1,2,4,8)}(n)>0$ for every $n\in \mathbb {N}$ and $m\in \{7,9,10,11,$ $12,13,14\}$, the conjecture is equivalent to showing that for every $n\in \mathbb {N}$, we have

1.4 $$\begin{aligned} s_{m,2(m-2),(1,2,4,8)}\left( 8(m-2)n + 15(m-4)^2\right) >0. \end{aligned}$$

We investigate the numbers $s_{r,M,{\varvec{\alpha }}}(n)$ by forming the generating function (setting $q:=e^{2\pi i \tau }$)

$$\begin{aligned} \Theta _{r,M,{\varvec{\alpha }}}(\tau ):=\sum _{n\ge 0} s_{r,M,{\varvec{\alpha }}}(n) q^{n}. \end{aligned}$$

It is well known that these functions are modular forms (see Lemma 2.1 for the precise statement). By the theory of modular forms, there is a natural decomposition

1.5 $$\begin{aligned} \Theta _{r,M,{\varvec{\alpha }}}=E_{r,M,{\varvec{\alpha }}} + f_{r,M,{\varvec{\alpha }}}, \end{aligned}$$

where $E_{r,M,{\varvec{\alpha }}}$ lies in the space spanned by Eisenstein series and $f_{r,M,{\varvec{\alpha }}}$ is a cusp form. In order to prove Theorem 1.2, we obtain in the special case $r=m$, $M=2(m-2)$, and ${\varvec{\alpha }}=(1,2,4,8)$ an explicit lower bound for the *n*-th Fourier coefficient $a_{r,M,{\varvec{\alpha }}}(n)$ of $E_{r,M,{\varvec{\alpha }}}$ in Corollary 4.2 and an explicit upper bound on the absolute value of the *n*-th Fourier coefficient $b_{r,M,{\varvec{\alpha }}}(n)$ of $f_{r,M,{\varvec{\alpha }}}$ in the proof of Theorem 1.2.

The paper is organized as follows. In Sect. 2, we recall properties of the theta functions $\Theta _{r,M,{\varvec{\alpha }}}$, the actions of certain operators on modular forms, the decomposition of modular forms into Eisenstein series and cusp forms, and evaluate certain Gauss sums. In Sect. 3, we investigate the growth of the theta functions toward all cusps and use this to compute the Eisenstein series component of the decomposition (1.5). The Fourier coefficients of the Eisenstein series components are then explicitly computed and lower bounds are obtained in Sect. 4. We complete the paper by obtaining upper bounds on the coefficients of the cuspidal part of the decomposition (1.5) and prove Theorem 1.2 in Sect. 5.

## Setup and Preliminaries

### Modularity of the Generating Functions

In this subsection, we consider the modularity properties of the theta functions $\Theta _{r,M,{\varvec{\alpha }}}$. To set notation, for $\Gamma _1(N)\subseteq \Gamma \subseteq {\textrm{SL}}_2(\mathbb {Z})$ ($N\in \mathbb {N}$) and a character $\chi$ modulo *N*, let $M_{k}(\Gamma ,\chi )$ be the space of modular forms of weight *k* with character $\chi$. In particular, an element *f* in this space satisfies, for ,

$$\begin{aligned} f\big |_k\gamma (\tau ):=(c\tau +d)^{-k}f(\gamma \tau )=\chi (d)f(\tau ). \end{aligned}$$

Setting $\Gamma _{N,L}:=\Gamma _0(N)\cap \Gamma _1(L)$, by [7, Theorem 2.4], we have the following.

#### Lemma 2.1

For ${\varvec{\alpha }}\in \mathbb {N}^4$, we have

$$\begin{aligned} \Theta _{r,M,{\varvec{\alpha }}} \in M_{2}\left( \Gamma _{4{\text {lcm}}({\varvec{\alpha }})M^2,M}, \left( \tfrac{\prod _{j=1}^4 \alpha _j}{\cdot }\right) \right) . \end{aligned}$$

### Operators on Non-holomorphic Modular Forms

For a translation-invariant function *f* with Fourier expansion (denoting $\tau =u+iv\in \mathbb {H}$)

$$\begin{aligned} f(\tau )=\sum _{n\ge 0} c_{f,v}(n) q^n, \end{aligned}$$

we define the *sieving operator* (*M*, $m\in \mathbb {N}$)

$$\begin{aligned} f\big |S_{M,m}(\tau ):=\sum _{\begin{array}{c} n\ge 0\\ n\equiv m\ \, \left( {\text {mod}} \, M \right) \end{array}} c_{f,v}(n) q^n. \end{aligned}$$

As usual, we also define the *V-operator* ($\delta \in \mathbb {N}$) by

$$\begin{aligned} f\big |V_{\delta }(\tau ):=\sum _{n\ge 0} c_{f,\delta v}(n)q^{\delta n}. \end{aligned}$$

We require the modularity properties of (non-holomorphic) modular forms under the operators $S_{M,m}$ and $V_d$. Arguing via commutator relations for matrices, a standard argument (for example, see the proof of [17, Lemma 2]), one obtains the following.

#### Lemma 2.2

Suppose that $k\in \mathbb {Z}$, $L,N\in \mathbb {N}$ with $L\mid N$, and *f* satisfies weight *k* modularity on $\Gamma _{N,L}$.

1. For $d\in \mathbb {N}$, the function $f|V_d$ satisfies weight *k* modularity on $\Gamma _{{\text {lcm}}(4,Nd),L}$.
2. For $m\in \mathbb {Z}$ and $M\in \mathbb {N}$, the function $f|S_{M,m}$ satisfies weight *k* modularity on $\Gamma _1(NM^2)$.

It is useful to determine the commutator relations between the *V*-operator and sieving.

#### Lemma 2.3

Let $m\in \mathbb {Z}$ and $M_1,M_2\in \mathbb {N}$ be given and set $d:=\gcd (M_1,M_2)$ and $\mu _j:=\frac{M_j}{d}$. Then for any translation-invariant function *f*, we have

$$\begin{aligned} f\big |V_{M_1}\big |S_{M_2,m}= {\left\{ \begin{array}{ll} f\big |S_{\mu _2,\overline{\mu }_1\frac{m}{d}}\big |V_{M_1}&{}\text {if }d\mid m,\\ 0&{}\text {otherwise}, \end{array}\right. } \end{aligned}$$

where $\bar{\mu }_1$ is the inverse of $\mu _1 \ \, \left( {\text {mod}} \, \mu _2 \right)$.

#### Proof

Recall that

$$\begin{aligned} f \left| S_{M_1,m}\right| V_{M_2}(\tau )&= \sum _{n\equiv m\ \, \left( {\text {mod}} \, M_1 \right) } c_{f,M_2v}(n)q^{M_2n},\\ f \left| V_{M_1}\right| S_{M_2,m}(\tau )&= \sum _{n} c_{f,M_1v}(n)q^{M_1n} \bigg | S_{M_2,m}(\tau )= \sum _{n\equiv m\ \, \left( {\text {mod}} \, M_2 \right) } \widetilde{c}_{f,v}(n)q^n\\&= \sum _{\begin{array}{c} n\equiv m \ \, \left( {\text {mod}} \, M_2 \right) \\ M_1\mid n \end{array}} c_{f,M_1v}\left( \frac{n}{M_1}\right) q^n, \end{aligned}$$

where

$$\begin{aligned} \widetilde{c}_{f,v}(n):={\left\{ \begin{array}{ll}c_{f,M_1v}\left( \frac{n}{M_1}\right) &{} \text {if } M_1 \mid n,\\ 0 &{} \text {otherwise}.\end{array}\right. } \end{aligned}$$

If $d=\gcd (M_1,M_2)\not \mid m$, then $n\equiv m\ \, \left( {\text {mod}} \, M_2 \right)$ and $M_1\mid n$ are not consistent, and hence $f |V_{M_1}|S_{M_2,m}$ vanishes identically.

We may hence assume that $d\mid m$ and we note that $\gcd (\mu _1,\mu _2)=1$, obtaining

$$\begin{aligned} f \left| V_{M_1}\right| S_{M_2,m}(\tau )&= \sum _{\begin{array}{c} \frac{n}{d} \equiv \frac{m}{d} \ \, \left( {\text {mod}} \, \mu _2 \right) \\ M_1\mid n \end{array}} c_{f,M_1v}\left( \frac{n}{M_1}\right) q^n\\&\overset{n \mapsto M_1n}{=}\ \sum _{n\equiv \bar{\mu }_1\frac{m}{d} \ \, \left( {\text {mod}} \, \mu _2 \right) } c_{f,M_1v}(n)q^{M_1n}\\&= f \left| S_{\mu _2,\bar{\mu }_1\frac{m}{d}}\right| V_{M_1}(\tau ). \end{aligned}$$

$$\square $$

### Decomposition Into Eisenstein Series and Cusp Forms

Comparing Fourier coefficients on both sides of (1.5), we have

2.1 $$\begin{aligned} s_{r,M,{\varvec{\alpha }}}(n)=a_{r,M,{\varvec{\alpha }}}(n)+b_{r,M,{\varvec{\alpha }}}(n). \end{aligned}$$

Theorem 1.2 is equivalent to showing (1.4) for *n* sufficiently large (with the restriction that $n\not \equiv 4\ \, \left( {\text {mod}} \, 16 \right)$ for $m=12$). Roughly speaking, the approach in this paper to proving (1.4) is to prove that for *n* sufficiently large with $n\equiv 15(m-4)^2\ \, \left( {\text {mod}} \, 8(m-2) \right)$ (noting the congruence conditions in (1.4))

$$\begin{aligned} a_{r,M,{\varvec{\alpha }}}(n)>|b_{r,M,{\varvec{\alpha }}}(n)|. \end{aligned}$$

To obtain an upper bound for $|b_{r,M,{\varvec{\alpha }}}(n)|$, we recall that Deligne [9] proved that for a normalized newform $f(\tau )=\sum _{n\ge 1} c_f(n)q^n$ of weight *k* on $\Gamma _0(N)$ with Nebentypus character $\chi$ (normalized so that $c_f(1)=1$), we have

2.2 $$\begin{aligned} |c_f(n)|\le \sigma _0(n) n^{\frac{k-1}{2}}, \end{aligned}$$

where $\sigma _k(n):=\sum _{d\mid n} d^k$. To obtain an explicit bound for $|c_f(n)|$ for arbitrary $f\in S_{k}(\Gamma _1(N))$, we combine (2.2) with a trick implemented by Blomer [5] and Duke [12]. For cusp forms $f,g\in S_{k}(\Gamma )$, we define the *Petersson inner product* by

$$\begin{aligned} \left<f,g\right>:=\frac{1}{[{\textrm{SL}}_2(\mathbb {Z}):\Gamma ]}\int _{\Gamma \backslash \mathbb {H}} f(\tau )\overline{g(\tau )} v^k \frac{dudv}{v^2}. \end{aligned}$$

Letting $\Vert f\Vert :=\sqrt{\left<f,f\right>}$ denote the *Petersson norm* of $f\in S_k(\Gamma )$, a bound for $|c_f(n)|$ in terms of $\Vert f\Vert$ may be obtained. Specifically, suppose that *f* is a cusp form *f* of weight $k\in \mathbb {N}$ on $\Gamma _{N,L}$ (with $L\mid N$) and character $\chi$ modulo *N*. Using Blomer’s method from [5], an explicit bound is obtained in [1, Lemma 4.1] for $|c_f(n)|$ as a function of *N*, *L*, and the Petersson norm $\Vert f\Vert$. Denoting by $\varphi$ Euler’s totient function, we recall a bound from the case $k=2$ below (see [1, (4.4)]).

#### Lemma 2.4

Suppose that $f\in S_{2}(\Gamma _{N,L},\chi )$ with $L\mid N$ and $\chi$ a character modulo *N*. Then, we have the inequality

$$\begin{aligned} \left| c_{f}(n)\right| \le 6.95\cdot 10^{18}\cdot \Vert f\Vert N^{1+2.5\cdot 10^{-6}}\prod _{p\mid N}\left( 1+\frac{1}{p}\right) ^{\frac{1}{2}}\varphi (L) n^{\frac{3}{5}}. \end{aligned}$$

By Lemma 2.4, in order to obtain an explicit bound for $|b_{r,M,{\varvec{\alpha }}}(n)|$, it remains to estimate $\Vert f_{r,M,{\varvec{\alpha }}}\Vert$, where $f_{r,M,{\varvec{\alpha }}}$ is the cusp form appearing in the decomposition in (1.5). An explicit bound for $\Vert f_{r,M,{\varvec{\alpha }}}\Vert$ was obtained in [16, Lemma 3.2]. To state the result, let ${\varvec{\alpha }}\in \mathbb {Z}^{\ell }$. For the quadratic form $Q=Q_{{\varvec{\alpha }}}$ given by

$$\begin{aligned} Q_{{\varvec{\alpha }}}({\varvec{x}}):=\sum _{j=1}^{\ell } \alpha _j x_j^2, \end{aligned}$$

we define the *level* and the *discriminant* of $Q_{{\varvec{\alpha }}}$ as

$$\begin{aligned} N_{{\varvec{\alpha }}}=4{\text {lcm}}({\varvec{\alpha }}),\qquad D_{{\varvec{\alpha }}}=2^{\ell }\prod _{j=1}^{\ell } \alpha _j. \end{aligned}$$

#### Lemma 2.5

Let $\ell \ge 4$ be even, ${\varvec{\alpha }}\in \mathbb {N}^{\ell }$, $r\in \mathbb {Z}$, and $M\in \mathbb {N}$. Then,

$$\begin{aligned} \Vert f_{r,M,{\varvec{\alpha }}}\Vert ^2\le & {} \frac{3^{2\ell -2}\left( \frac{\ell }{2}-2\right) !}{2^{\frac{\ell }{2}-3}\pi ^{\ell }} \frac{M^{2\ell -4}N_{{\varvec{\alpha }}}^{\ell -2}}{\prod _{p\mid M^2N_{{\varvec{\alpha }}}}\left( 1-p^{-2}\right) }\\{} & {} \times \sum _{\delta \mid M^2N_{{\varvec{\alpha }}}}\varphi \left( \frac{M^2N_{{\varvec{\alpha }}}}{\delta }\right) \varphi (\delta )\frac{M^2N_{{\varvec{\alpha }}}}{\delta }\left( \frac{\gcd (M^2,\delta )}{M^2}\right) ^{\ell } \\{} & {} \times \sum _{m=0}^{\frac{\ell }{2}-2} \frac{(2\pi )^{-m}}{\left( \frac{\ell }{2}-2-m\right) !}(\ell -m-2)! \left( \frac{9}{ D_{{\varvec{\alpha }}}}(\ell -m-1)\frac{M^2N_{{\varvec{\alpha }}}}{\pi } +\ell ^2 \right) . \end{aligned}$$

### Gauss Sums

Define the *generalized quadratic Gauss sum* ($a,b\in \mathbb {Z}$, $c\in \mathbb {N}$)

$$\begin{aligned} G(a,b;c):=\sum _{\ell \ \, \left( {\text {mod}} \, c \right) }e^{\frac{2\pi i}{c}\left( a\ell ^2+b\ell \right) }. \end{aligned}$$

Background information and many properties of these sums may be found in [2]. To state the properties that we require, for *d* odd, we define

$$\begin{aligned} \varepsilon _d:={\left\{ \begin{array}{ll} 1&{}\text {if }d\equiv 1\ \, \left( {\text {mod}} \, 4 \right) ,\\ i&{}\text {if }d\equiv 3\ \, \left( {\text {mod}} \, 4 \right) ,\end{array}\right. } \end{aligned}$$

and we write $[a]_{b}$ for the inverse of *a* modulo *b* if $\gcd (a,b)=1$.

#### Lemma 2.6

For $a,b\in \mathbb {Z}$ and $c,d\in \mathbb {N}$, the following hold.

1. If $\gcd (a,c)\not \mid b$, then $G(a,b;c)=0$, while if $\gcd (a,c)\mid b$ then
  $$\begin{aligned} G(a,b;c)=\gcd (a,c) G\left( \frac{a}{\gcd (a,c)},\frac{b}{\gcd (a,c)};\frac{c}{\gcd (a,c)}\right) . \end{aligned}$$
2. If $\gcd (a,c)=1$ and *c* is odd, then
  $$\begin{aligned} G(a,b;c) = \varepsilon _c \sqrt{c} \left( \frac{a}{c}\right) e^{-\frac{2\pi i [4a]_c b^2}{c}}. \end{aligned}$$
3. If $$\gcd (c,d)=1$$, then
  $$\begin{aligned} G(a,b;cd)=G(ac,b;d)G(ad,b;c). \end{aligned}$$
4. If $\gcd (a,c)=1$, $4\mid c$, and *b* is odd, then $G(a,b;c)=0$.
5. If *a* is odd, *b* is even, and $r\in \mathbb {N}_{\ge 2}$, then
  $$\begin{aligned} G\left( a,b;2^r\right) = 2^{\frac{r}{2}} (1+i) \left( \frac{-2^{r}}{a}\right) \varepsilon _a e^{\frac{2\pi i}{2^{r}} \left( -[a]_{2^{r}} \frac{b^2}{4}\right) }. \end{aligned}$$

We require an explicit evaluation of certain Gauss sums that naturally occur in the study of theta functions (see Lemma 3.1 below). Throughout the paper, for $k,M\in \mathbb {N}$, and $r\in \mathbb {Z}$ with ${\text{ ord }}_2(r)\le {\text{ ord }}_2(M)$, we write $M=2^{\mu }M_0$, $r=2^{\varrho }r_0$ (with $\varrho \le \mu$), and $k=2^{\kappa }k_0$ with $M_0$, $r_0$, and $k_0$ odd. We furthermore set $g_0:=\gcd (M_0,k_0)$ and $g_1:=\gcd (g_0,\frac{k_0}{g_0})$.

#### Lemma 2.7

Suppose that $h\in \mathbb {Z}$, $k\in \mathbb {N}$ with $\gcd (h,k)=1$, $\ell \in \mathbb {N}_0$, $r\in \mathbb {Z}$, $M\in \mathbb {N}$ with $\gcd (M,r)\in \{1,2,4\}$, and $\varrho \le \mu$.

1. If $g_1\ne 1$ or $\varrho < \min (\mu ,\kappa -\ell -\mu )-1$, then
  $$\begin{aligned} G\left( 2^{\ell }h M^2,2^{\ell +1}hrM;k\right) =0. \end{aligned}$$
2. Suppose that $g_1=1$ and $\varrho \ge \min (\mu , \kappa -\ell -\mu )-1$. Setting $\delta :=\min (\ell +2\varrho ,\kappa )$, we then have
  $$\begin{aligned}{} & {} e^{\frac{2\pi i 2^{\ell }hr^2}{k}}G\left( 2^{\ell } h M^2,2^{\ell +1}hrM;k\right) \\{} & {} \quad =\sqrt{k g_0}{\left\{ \begin{array}{ll} 2^{\frac{\kappa }{2}} \varepsilon _{\frac{k_0}{g_0}} \left( \frac{2^{\ell +\kappa } h g_0}{\frac{k_0}{g_0}}\right) e^{\frac{2\pi i hr_0^2}{2^{\kappa -\delta }g_0} 2^{\ell +2\varrho -\delta } \left[ \frac{k_0}{g_0}\right] _{2^{\kappa -\delta }g_0}}&{}\begin{array}{l}\text {if }\kappa \le \ell +2\mu \\ \quad \text {or }\kappa =\ell +2\mu +1\text { and }\varrho =\mu -1,\end{array}\\ 2^{\frac{\ell +2\mu }{2}}(1+i) \varepsilon _{hg_0} \left( \frac{-2^{\ell +\kappa }\frac{k_0}{g_0}}{hg_0}\right) e^{\frac{2\pi i hr_0^2}{g_0}\left[ 2^{\kappa -\ell -2\mu }\frac{k_0}{g_0}\right] _{g_0}}&{}\text {if }\kappa \ge \ell +2\mu +2\text { and }\varrho =\mu ,\\ 0&{}\text {otherwise.} \end{array}\right. } \end{aligned}$$

#### Proof

We evaluate *G*(*a*, *b*; *c*) for $a:= 2^{\ell } h M^2$, $b:=2^{\ell +1}hrM$, and $c:=k$. By Lemma 2.6 (1), $G(a,b;c)=0$ unless $\gcd (a,c)\mid b$. Hence, we first compute, using the fact that $\gcd (h,k)=1$, $\gcd (\frac{M_0}{g_0},\frac{k_0}{g_0})=1$, and $\frac{k_0}{g_0}$ is odd,

2.3 $$\begin{aligned} \gcd (a,c) = 2^{\min (\ell +2\mu ,\kappa )}g_0g_2, \end{aligned}$$

where $$g_2:=\gcd (M_0,\frac{k_0}{g_0})$$.

1. A direct calculation gives that $\gcd (a,c)\mid b$ if and only if $g_1=1$ and $\varrho \ge \min (\mu ,\kappa -\ell -\mu )-1$, which implies the claim by Lemma 2.6 (1).
2. Set $\gamma :=\min (\ell +2\mu ,\kappa )$. Note that $\gamma \le \ell +\mu +\varrho +1$. From the calculation yielding (1), we see that $g_1=1$ implies $g_2=1$. Plugging $g_1=g_2=1$ into (2.3) yields $\gcd (a,c)=2^{\gamma }g_0$ and it is not hard to see that $\gcd (a,c)\mid b$. Therefore, Lemma 2.6 (1),(3) implies that
  $$\begin{aligned} G(a,b;c)= & {} 2^{\gamma } g_0G\left( 2^{\ell +2\mu +\kappa -2\gamma } hM_0 \frac{M_0}{g_0}, 2^{\ell +\mu +\varrho +1-\gamma } hr_0\frac{M_0}{g_0}; \frac{k_0}{g_0}\right) \\{} & {} \times G\left( 2^{\ell +2\mu -\gamma } h M_0 \frac{M_0}{g_0}\frac{k_0}{g_0}, 2^{\ell +\mu +\varrho +1-\gamma } hr_0\frac{M_0}{g_0}; 2^{\kappa -\gamma }\right) . \end{aligned}$$
  Since $\frac{k_0}{g_0}$ is odd, we use Lemma 2.6 (2) to evaluate the first Gauss sum, yielding, after simplification,
  2.4 $$\begin{aligned} G(a,b;c)&=2^{\gamma } \varepsilon _{\frac{k_0}{g_0}} \sqrt{k_0 g_0} \left( \frac{2^{\ell +\kappa } h g_0}{\frac{k_0}{g_0}}\right) e^{-\frac{2\pi i hr_0^2}{\frac{k_0}{g_0}} 2^{\ell +2\varrho }\left[ 2^{\kappa }g_0\right] _{\frac{k_0}{g_0}}}\nonumber \\&\quad \times G\left( 2^{\ell +2\mu -\gamma } h M_0 \frac{M_0}{g_0}\frac{k_0}{g_0}, 2^{\ell +\mu +\varrho +1-\gamma } hr_0\frac{M_0}{g_0}; 2^{\kappa -\gamma }\right) . \end{aligned}$$

It remains to evaluate the final Gauss sum in (2.4). We use Lemma 2.6 (4) and Lemma 2.6 (5) to obtain

2.5 $$\begin{aligned}&G\left( 2^{\ell +2\mu -\gamma } h M_0 \frac{M_0}{g_0}\frac{k_0}{g_0}, 2^{\ell +\mu +\varrho +1-\gamma } hr_0\frac{M_0}{g_0}; 2^{\kappa -\gamma }\right) \nonumber \\&={\left\{ \begin{array}{ll} 1 &{}\text {if }\kappa \le \ell +2\mu ,\\ 2&{}\text {if }\kappa =\ell +2\mu +1,\, \varrho =\mu -1,\\ 2^{\frac{\kappa -\ell -2\mu }{2}} (1+i) \left( \frac{-2^{\ell +\kappa }}{h M_0 \frac{M_0}{g_0}\frac{k_0}{g_0}}\right) \varepsilon _{h M_0 \frac{M_0}{g_0}\frac{k_0}{g_0}} e^{-\frac{2\pi i hr_0^2}{2^{\kappa -\ell -2\mu }}\left[ k_0\right] _{2^{\kappa -\ell -2\mu }} }&{}\text {if }\kappa \ge \ell +2\mu +2,\, \varrho =\mu ,\\ 0&{}\text {otherwise}. \end{array}\right. } \end{aligned}$$

Plugging (2.5) into (2.4) and then simplifying yields that *G*(*a*, *b*; *c*) equals

$$\begin{aligned}&\varepsilon _{\frac{k_0}{g_0}}\sqrt{kg_0} \left( \tfrac{h g_0}{\frac{k_0}{g_0}}\right) {\left\{ \begin{array}{ll} 2^{\frac{\kappa }{2}} \left( \tfrac{2^{\ell +\kappa } }{\frac{k_0}{g_0}}\right) e^{-\frac{2\pi i hr_0^2}{\frac{k_0}{g_0}}2^{\ell +2\varrho }\left[ 2^{\kappa }g_0\right] _{\frac{k_0}{g_0}}} &{}\begin{array}{l}\text {if }\kappa \le \ell +2\mu \\ \quad \text {or }\kappa =\ell +2\mu +1,\, \varrho =\mu -1,\end{array}\\ 2^{\frac{\ell +2\mu }{2}}(1+i) \varepsilon _{h M_0 \frac{M_0}{g_0}\frac{k_0}{g_0}} \left( \frac{-2^{\ell +\kappa }}{h M_0 \frac{M_0}{g_0}\frac{k_0}{g_0}}\right) \left( \tfrac{2^{\ell +\kappa } }{\frac{k_0}{g_0}}\right) &{}\text {if }\kappa \ge \ell +2\mu +2,\, \varrho =\mu ,\\ \times e^{-\frac{2\pi i hr_0^2}{\frac{k_0}{g_0}} 2^{\ell +2\mu }\left[ 2^{\kappa }g_0\right] _{\frac{k_0}{g_0}} }e^{-\frac{2\pi i hr_0^2}{2^{\kappa -\ell -2\mu }}\left[ k_0\right] _{2^{\kappa -\ell -2\mu }}}&{}\\ 0&{}\text {otherwise}. \end{array}\right. } \end{aligned}$$

To obtain the claim, we multiply by $e^{\frac{2\pi i 2^{\ell }hr^2}{k}}$ and simplify by using the Chinese Remainder Theorem to combine the exponentials. For example, if $\kappa \le \ell +2\mu$ or ($\kappa =\ell +2\mu +1$ and $\varrho =\mu -1$), then the exponential becomes

$$\begin{aligned} e^{\frac{2\pi i hr_0^2}{2^{\kappa -\delta }k_0} 2^{\ell +2\varrho -\delta }\left( 1-2^{\kappa } g_0 \left[ 2^{\kappa }g_0\right] _{\frac{k_0}{g_0}}\right) }. \end{aligned}$$

Since $\gcd (g_0,\frac{k_0}{g_0})=g_1=1$ and $k_0$ is odd, to determine $1-2^{\kappa }g_0 \left[ 2^{\kappa }g_0\right] _{\frac{k_0}{g_0}} \ \, \left( {\text {mod}} \, 2^{\kappa -\delta }k_0 \right)$ the Chinese Remainder Theorem implies that it suffices to compute

$$\begin{aligned} 1-2^{\kappa }g_0 \left[ 2^{\kappa }g_0\right] _{\frac{k_0}{g_0}}&\equiv 1 \ \, \left( {\text {mod}} \, g_0 \right) ,\qquad 1-2^{\kappa }g_0 \left[ 2^{\kappa }g_0\right] _{\frac{k_0}{g_0}}\equiv 0 \ \, \left( {\text {mod}} \, \frac{k_0}{g_0} \right) ,\\ 1-2^{\kappa }g_0 \left[ 2^{\kappa }g_0\right] _{\frac{k_0}{g_0}}&\equiv 1 \ \, \left( {\text {mod}} \, 2^{\kappa -\delta } \right) . \end{aligned}$$

Thus,

$$\begin{aligned} 1-2^{\kappa }g_0 \left[ g_0\right] _{\frac{k_0}{g_0}}\equiv \frac{k_0}{g_0}\left[ \frac{k_0}{g_0}\right] _{2^{\kappa -\delta }g_0} \ \, \left( {\text {mod}} \, 2^{\kappa -\delta }k_0 \right) . \end{aligned}$$

So the exponential simplifies in this case as $e^{\frac{2\pi i hr_0^2}{2^{\kappa -\delta }g_0}2^{\ell +2\varrho -\delta }[\frac{k_0}{g_0}]_{2^{\kappa -\delta }g_0}}$.

The remaining case $\kappa \ge \ell +2\mu +2$ and $\varrho =\mu$ follows by a similar but longer and more tedious calculation. $\square$

## Growth Toward the Cusps of Certain Modular Forms

In this section, we determine the growth toward the cusps of theta functions $\Theta _{r,M,{\varvec{\alpha }}}$ and certain (non-holomorphic) Eisenstein series. The purpose of this calculation is to compare the growth in order to determine the unique Eisenstein series $E_{r,M,{\varvec{\alpha }}}$ in (1.5) whose growth toward the cusps matches that of the theta function.

### Growth of the Theta Functions at the Cusps

In order to obtain the Eisenstein series, we determine the growth of $\Theta _{r,M,{\varvec{\alpha }}}$ toward all of the cusps, which follows by a straightforward calculation.

#### Lemma 3.1

Let $m\in \mathbb {N}_{\ge 3}$ and ${\varvec{\alpha }}\in \mathbb {N}^4$ be given. For $h\in \mathbb {Z}$ and $k\in \mathbb {N}$ with $\gcd (h,k)=1$, we have

$$\begin{aligned}{} & {} -\lim _{z\rightarrow 0^+} z^{2}\Theta _{r,M,{\varvec{\alpha }}} \left( \frac{h}{k}+\frac{iz}{k}\right) \\{} & {} \quad =-\frac{1}{4k^2M^4\prod _{j=1}^4\sqrt{\alpha _j}} \prod _{j=1}^4 e^{\frac{2\pi i r^2h\alpha _j}{k}} G \left( h\alpha _jM^2,2hr\alpha _jM;k\right) . \end{aligned}$$

#### Proof

We have

3.1 $$\begin{aligned} \Theta _{r,M,{\varvec{\alpha }}}(\tau )=\sum _{\begin{array}{c} {\varvec{x}}\in \mathbb {Z}^d\\ x_j\equiv r\ \, \left( {\text {mod}} \, M \right) \end{array}} q^{\sum _{j=1}^{4}\alpha _jx_j^2} =\prod _{j=1}^4\vartheta (r,M;2M\alpha _j\tau ), \end{aligned}$$

where

$$\begin{aligned} \vartheta (r,M;\tau ):=\sum _{n\equiv r\ \, \left( {\text {mod}} \, M \right) }q^{\frac{n^2}{2M}}. \end{aligned}$$

By definition,

$$\begin{aligned} \vartheta \left( r,M;2M\alpha _j\left( \frac{h}{k}+\frac{iz}{k}\right) \right) =\sum _{\begin{array}{c} n\equiv r\ \, \left( {\text {mod}} \, M \right) \end{array}} e^{2\pi i \alpha _j n^2\left( \frac{h}{k}+\frac{iz}{k}\right) }. \end{aligned}$$

Write $n=r+M\alpha +Mk\ell$ ($\alpha \ \, \left( {\text {mod}} \, k \right) ,\ell \in \mathbb {Z}$) to obtain that this equals

$$\begin{aligned}{} & {} \sum _{\alpha \ \, \left( {\text {mod}} \, k \right) }e^{2\pi i \alpha _j(r+M\alpha )^2\frac{h}{k}} {\sum _{\ell \in \mathbb {Z}}e^{-2\pi (r+M\alpha +Mk\ell )^2\frac{\alpha _j}{k}z}}\\{} & {} \quad =\sum _{\alpha \ \, \left( {\text {mod}} \, k \right) }e^{2\pi i \alpha _j(r+M\alpha )^2\frac{h}{k}}\vartheta (r+M\alpha ,Mk;2M\alpha _j iz). \end{aligned}$$

We now recall the modular inversion formula (see [19, (2.4)])

$$\begin{aligned} \vartheta \left( r,M;-\frac{1}{\tau }\right) =M^{-\frac{1}{2}}(-i\tau )^\frac{1}{2} \sum _{\nu \ \, \left( {\text {mod}} \, M \right) } e^{\frac{2\pi i \nu k}{M}}\vartheta (\nu ,M;\tau ). \end{aligned}$$

We use this with $\tau =\frac{i}{2M\alpha _jz}$, $r\mapsto r+M\alpha$, $M\mapsto Mk$ to obtain that

$$\begin{aligned}{} & {} \vartheta (r+M\alpha ,Mk;2M\alpha _j iz)\\{} & {} \quad =(Mk)^{-\frac{1}{2}}\left( \frac{1}{2M\alpha _j z}\right) ^\frac{1}{2} \sum _{\nu \ \, \left( {\text {mod}} \, Mk \right) }e^{\frac{2\pi i }{Mk}(r+M\alpha )\nu } \vartheta \left( \nu ,Mk;\frac{i}{2M\alpha _j z}\right) . \end{aligned}$$

Thus,

$$\begin{aligned} \vartheta \left( r,M;2M\alpha _j\left( \frac{h}{k}+\frac{iz}{k}\right) \right)= & {} \frac{1}{M}\sqrt{\frac{1}{2k\alpha _jz}}\sum _{\alpha \ \, \left( {\text {mod}} \, k \right) }e^{2\pi i \alpha _j(r+M\alpha )^2\frac{h}{k}} \\ {}{} & {} \times \sum _{\nu \ \, \left( {\text {mod}} \, Mk \right) }e^{\frac{2\pi i }{Mk}(r+M\alpha )\nu } \vartheta \left( \nu ,Mk;\frac{i}{2M\alpha _jz}\right) . \end{aligned}$$

Now assume that $z\in \mathbb {R}^+$ and let $z\rightarrow 0^+$. The contribution that is not exponentially decaying comes from $\nu =0$ and gives

$$\begin{aligned} \lim _{z\rightarrow 0^+}\sqrt{z}\vartheta \left( r,M;2M\alpha _j\left( \frac{h}{k}+\frac{iz}{k}\right) \right) = \frac{1}{M}\sqrt{\frac{1}{2k\alpha _j}} {\sum _{\alpha \ \, \left( {\text {mod}} \, k \right) }e^{2\pi i \alpha _j(r+M\alpha )^2\frac{h}{k}}}. \end{aligned}$$

Note that

$$\begin{aligned} \sum _{\alpha \ \, \left( {\text {mod}} \, k \right) }e^{2\pi i \alpha _j(r+M\alpha )^2\frac{h}{k}}&=e^{\frac{2\pi i \alpha _jr^2h}{k}} \sum _{\alpha \ \, \left( {\text {mod}} \, k \right) }e^{\frac{2\pi i }{k} \left( \alpha _jM^2\alpha ^2+2r\alpha _jM\alpha \right) h}\\&= e^{\frac{2\pi i \alpha _jr^2h}{k}} G\left( h\alpha _jM^2,2hr\alpha _jM;k\right) . \end{aligned}$$

Plugging back into (3.1) yields the claim. $\square$

We next use Lemma 2.7 to evaluate the right-hand side of Lemma 3.1. Since the theta function $\Theta _{r,M,{\varvec{\alpha }}}$ only depends on *r* modulo *M*, we may assume without loss of generality that

$$\begin{aligned} \varrho ={\text{ ord }}_2(r)\le {\text{ ord }}_2(M)=\mu \end{aligned}$$

by replacing *r* with $r+M$ in Lemma 3.1 if $\varrho >\mu$. A direct calculation gives the following.

#### Corollary 3.2

Suppose that $h\in \mathbb {Z}$ and $k\in \mathbb {N}$ with $\gcd (h,k)=1$, ${\varvec{\alpha }}=(1,2,4,8)$, $r\in \mathbb {Z}$, and $M\in \mathbb {N}$ with $\gcd (M,r)\in \{1,2,4\}$ and ${\text{ ord }}_2(r)\le {\text{ ord }}_2(M)$. If $g_1\ne 1$ or $\varrho <\min (\mu ,k-\ell -\mu )-1$, then

$$\begin{aligned} -\lim _{z\rightarrow 0^+} z^{2}\Theta _{r,M,{\varvec{\alpha }}} \left( \frac{h}{k}+\frac{iz}{k}\right) =0. \end{aligned}$$

If $g_1=1$ and $\varrho \ge \min (\mu ,k-\ell -\mu )-1$, then, setting $\delta _0:=\min (\kappa ,2\varrho )$,

$$\begin{aligned}&-\lim _{z\rightarrow 0^+} z^{2}\Theta _{r,M,{\varvec{\alpha }}}\left( \frac{h}{k}+\frac{iz}{k}\right) \\&\quad = {\left\{ \begin{array}{ll} - \frac{2^{2\kappa -4\mu -5}}{M_0^4} g_0^2 e^{\frac{2\pi i hr_0^2}{2^{\kappa -\delta _0}g_0} 2^{2\varrho -\delta _0} 15 \left[ \frac{k_0}{g_0}\right] _{2^{\kappa -\delta _0}g_0}}&{}\text {if }\kappa \le 2\mu ,\\ &{}\quad \text {or }\kappa =2\mu +1\text { and }\varrho =\mu -1,\\ \frac{g_0^2}{M_0^4} e^{\frac{2\pi i hr_0^2}{g_0} 15\left[ 2^{\kappa -2\mu }\frac{k_0}{g_0}\right] _{g_0}} &{}\text {if }\kappa \ge 2\mu +5\text { and }\varrho =\mu ,\\ 0&{}\text {otherwise.} \end{array}\right. } \end{aligned}$$

#### Remark

Although the right-hand side of Corollary 3.2 splits into a number of cases, we obtain an explicit element of the cyclotomic field $\mathbb {Q}(\zeta _{2^{j}g_0})$ for some $j\in \mathbb {N}_0$, where $\zeta _{\nu }:=e^{\frac{2\pi i}{\nu }}$. To use Corollary 3.2 for practical purposes, one can evaluate the right-hand side of Corollary 3.2 with a computer as an element of $\mathbb {Q}(\zeta _\nu )\cong \mathbb {Q}[x]/\left<f_{\nu }\right>$, where $f_\nu$ is the minimal polynomial of $\zeta _\nu$ over $\mathbb {Q}$, which is well known to be

$$\begin{aligned} f_\nu (x)=\prod _{\begin{array}{c} 1\le k\le \nu \\ \gcd (k,\nu )=1 \end{array}}\left( x-\zeta _\nu ^k\right) = \prod _{d\mid \nu } \left( x^d-1\right) ^{\mu \left( \frac{\nu }{d}\right) }. \end{aligned}$$

Here, $\mu$ denotes the *Möbius* $\mu$-*function*.

### Growth of Eisenstein Series Toward the Cusps

The goal of this section is to obtain the growth of certain weight two Eisenstein series toward the cusps. These are formed by applying certain sieving and *V*-operators to the (non-holomorphic but modular) weight two Eisenstein series

$$\begin{aligned} \widehat{E}_2(\tau ):=E_2(\tau )-\frac{3}{\pi v}, \qquad \text { where }\qquad E_2(\tau ):=1-24\sum _{n\ge 1} \sigma (n) q^n \end{aligned}$$

with $\sigma (n):=\sigma _1(n)$. In light of Lemma 2.3, we may furthermore always assume without loss of generality that sieving is applied before the *V*-operator. The growth toward the cusps of such functions is given in the following lemma.

#### Lemma 3.3

Let $m\in \mathbb {Z}$ and $M_1,M_2\in \mathbb {N}$. Then, for $h\in \mathbb {Z}$ and $k\in \mathbb {N}$ with $\gcd (h,k)=1$, we have

$$\begin{aligned}{} & {} -\lim _{z\rightarrow 0^+}z^2\widehat{E}_2\big |S_{M_1,m}\big |V_{M_2}\left( \frac{h}{k}+\frac{iz}{k}\right) \\{} & {} \quad = \frac{1}{M_1^3M_2^2}\sum _{j\ \, \left( {\text {mod}} \, M_1 \right) } \gcd \left( hM_1M_2+jk,M_1k\right) ^2 \zeta _{M_1}^{-jm}. \end{aligned}$$

#### Proof

For a translation-invariant function *f*, we use the presentation

$$\begin{aligned} f|S_{M_1,m}(\tau )=\frac{1}{M_1} \sum _{j=0}^{M_1-1} \zeta _{M_1}^{-jm}f\left( \tau +\frac{j}{M_1} \right) . \end{aligned}$$

Applying $V_{M_2}$ to this yields

$$\begin{aligned} f|S_{M_1,m}\big |V_{M_2}(\tau )=\frac{1}{M_1} \sum _{j=0}^{M_1-1} \zeta _{M_1}^{-jm}f\left( M_2\tau +\frac{j}{M_1} \right) . \end{aligned}$$

Plugging in $f=\widehat{E}_2$ and using the weight two modularity of $\widehat{E}_2$, the claim follows by a standard calculation. $\square$

## Eisenstein Series Component

In this section, we determine the Eisenstein series component $E_{r,M,{\varvec{\alpha }}}$ in (1.5).

### Proposition 4.1

For $n\in \mathbb {N}$, we have the following.

1. For $m=7$, we have $a_{7,10,(1,2,4,8)}(n)=0$ unless $n\equiv 15\ \, \left( {\text {mod}} \, 40 \right)$, in which case we have
  $$\begin{aligned} a_{7,10,(1,2,4,8)}(n) = \frac{1}{240}\left( \sigma (n)-\sigma \left( \frac{n}{5}\right) \right) . \end{aligned}$$
2. For $m=9$, we have $a_{9,14,(1,2,4,8)}(n)=0$ unless $n\equiv 39\ \, \left( {\text {mod}} \, 56 \right)$, in which case we have
  $$\begin{aligned} a_{9,14,(1,2,4,8)}(n) = \frac{1}{672} \sigma (n). \end{aligned}$$
3. For $m=10$, we have $a_{10,16,(1,2,4,8)}(n)=0$ unless $n\equiv 28\ \, \left( {\text {mod}} \, 64 \right)$, in which case we have
  $$\begin{aligned} a_{10,16,(1,2,4,8)}(n) = \frac{1}{256} \sigma \left( \frac{n}{4}\right) . \end{aligned}$$
4. For $m=11$, we have $a_{11,18,(1,2,4,8)}(n)=0$ unless $n\equiv 15\ \, \left( {\text {mod}} \, 72 \right)$, in which case we have
  $$\begin{aligned} a_{11,18,(1,2,4,8)}(n) = \frac{1}{1728} \sigma (n). \end{aligned}$$
5. For $m=12$, we have $a_{12,20,(1,2,4,8)}(n)=0$ unless $80\mid n$, in which case we have
  $$\begin{aligned} a_{12,20,(1,2,4,8)}(n)= & {} \frac{1}{120} \left( \sigma \left( \frac{n}{16}\right) -\sigma \left( \frac{n}{32}\right) - \sigma \left( \frac{n}{80}\right) +\sigma \left( \frac{n}{160}\right) \right. \\ {}{} & {} \left. +8\sigma \left( \frac{n}{256}\right) -32\sigma \left( \frac{n}{512}\right) \right. \\{} & {} \left. -8\sigma \left( \frac{n}{1280}\right) +32\sigma \left( \frac{n}{2560}\right) \right) . \end{aligned}$$
6. For $m=13$, we have $a_{13,22,(1,2,4,8)}(n)=0$ unless $n\equiv 71\ \, \left( {\text {mod}} \, 88 \right)$, in which case we have
  $$\begin{aligned} a_{13,22,(1,2,4,8)}(n) = \frac{1}{2640} \sigma (n). \end{aligned}$$
7. For $m=14$, we have
  $$\begin{aligned} a_{14,24,(1,2,4,8)}(n) = {\left\{ \begin{array}{ll} \frac{1}{768} \left( \sigma \left( \frac{n}{4}\right) -\sigma \left( \frac{n}{12}\right) \right) &{}\text {if }n\equiv 60\ \, \left( {\text {mod}} \, 96 \right) ,\\ 0&{}\text {otherwise}. \end{array}\right. } \end{aligned}$$

### Proof

(1) By comparing Fourier coefficients, we see that the identity is equivalent to

4.1 $$\begin{aligned} E_{7,10,(1,2,4,8)}=-\frac{1}{5760}E_2\big |\left( 1-V_5\right) \big |S_{40,15}. \end{aligned}$$

Lemma 2.1 and (1.5) give that

$$\begin{aligned} E_{7,10,\left( 1,2,4,8\right) } \in M_2\Big (\Gamma _{3200,10}\Big ), \end{aligned}$$

while Lemma 2.2 implies that

$$\begin{aligned} E_2\big |\left( 1-V_5\right) \big |S_{40,15}\in M_2\left( \Gamma _1\left( 1600\right) \right) . \end{aligned}$$

Enumerating the cusps of $\Gamma _1(3200)$ (see [10, Proposition 3.8.3]), we then use a computer together with Lemma 3.3 and Corollary 3.2 to verify that the growth toward every cusp of both sides of (4.1) agrees, yielding the claim.

To see this in more details note that by [10, Proposition 3.8.3], two cusps $\frac{a}{c}$ and $\frac{\alpha }{\gamma }$ are equivalent modulo the action of $\Gamma _1(N)$ if and only if there exists $j\in \mathbb {Z}$ such that $(\alpha ,\gamma )\equiv \pm (a+jc,c)\ \, \left( {\text {mod}} \, N \right)$ (for some choice of ±). As in [10, p. 102], by taking $d:=\gcd (c,N)$, we may write a set of representatives of the inequivalent cusps in the form $\frac{a}{d\gamma }$ with $d\mid N$, *a* running modulo *d* with $\gcd (a,d)=1$, and $1\le \gamma \le \lceil \frac{N}{2d}\rceil$ with $\gcd (\gamma ,\frac{N}{d})=1$. Since both sides of (4.1) are elements of $M_2(\Gamma _1(3200))$, we thus need to compute the constant term at every cusp $\frac{h}{k}$ with $h,k\in \mathbb {Z}$, $\gcd (h,k)=1$, and $k=d\gamma$ with $d\mid 3200$ and $1\le \gamma \le \frac{1600}{d}$ with $\gcd (\gamma ,\frac{3200}{d})=1$. For each such representative $\frac{h}{k}$ of the cusps of $\Gamma _1(3200)$, we use Lemma 3.3 together with a computer to evaluate

$$\begin{aligned} -\frac{1}{5760} \lim _{z\rightarrow 0^+} z^2\left( E_2\big |S_{8,3}\big |V_5\left( \frac{h}{k}+\frac{iz}{k}\right) -E_2\big |S_{40,15}\left( \frac{h}{k}+\frac{iz}{k}\right) \right) \end{aligned}$$

as an element of $\mathbb {Q}(\zeta _{40})$. Comparing this with Corollary 3.2 in the case $r=7$ and $M=10$, we then verify with a computer that

4.2 $$\begin{aligned}{} & {} -\frac{1}{5760}\lim _{z\rightarrow 0^+}z^2\left( E_2\big |S_{8,3}\big |V_5\left( \frac{h}{k}+\frac{iz}{k}\right) - E_2\big |S_{40,15}\left( \frac{h}{k}+\frac{iz}{k}\right) \right) \nonumber \\{} & {} \quad = -\lim _{z\rightarrow 0^+} z^{2}\Theta _{7,10,{\varvec{\alpha }}} \left( \frac{h}{k}+\frac{iz}{k}\right) . \end{aligned}$$

Since $f_{7,10,(1,2,4,8)}$ is a cusp form, we have

$$\begin{aligned} -\lim _{z\rightarrow 0^+} z^{2}f_{7,10,{\varvec{\alpha }}} \left( \frac{h}{k}+\frac{iz}{k}\right) =0 \end{aligned}$$

and hence

$$\begin{aligned} -\lim _{z\rightarrow 0^+} z^{2}\Theta _{7,10,{\varvec{\alpha }}} \left( \frac{h}{k}+\frac{iz}{k}\right) =-\lim _{z\rightarrow 0^+} z^{2}E_{7,10,{\varvec{\alpha }}} \left( \frac{h}{k}+\frac{iz}{k}\right) . \end{aligned}$$

Therefore, (4.2) implies that

$$\begin{aligned} E_{7,10,(1,2,4,8)}+\frac{1}{5760} E_2\big |(1-V_5) S_{40,15} \end{aligned}$$

vanishes toward all cusps, and is hence a cusp form. Since it is also in the subspace of Eisenstein series, it is orthogonal to all cusp forms and therefore vanishes, implying (4.1), and hence the claim.

For the remaining cases, the argument is similar, but we provide the identities analogous to (4.1) for the convenience of the reader.

(2) The claim is equivalent to
  $$\begin{aligned} E_{9,14,(1,2,4,8)}=-\frac{1}{16128}E_2\big |S_{56,39}. \end{aligned}$$
(3) The claim is equivalent to
  $$\begin{aligned} E_{10,16,(1,2,4,8)}=-\frac{1}{6144}E_2 \big |S_{16,7}\big |V_4. \end{aligned}$$
(4) The claim is equivalent to
  $$\begin{aligned} E_{11,18,(1,2,4,8)}=-\frac{1}{41472}E_2 \big |S_{72,15}. \end{aligned}$$
(5) The claim is equivalent to
  $$\begin{aligned} E_{12,20,(1,2,4,8)}=-\frac{1}{2880}E_2\big |\left( S_{5,0}-V_5\right) \big |\left( 1- V_{2}+ 8V_{16}-32V_{32}\right) \big |V_{16}. \end{aligned}$$
(6) The claim is equivalent to
  $$\begin{aligned} E_{13,22,(1,2,4,8)}=-\frac{1}{63360}E_2 \big |S_{88,71}. \end{aligned}$$
(7) The claim is equivalent to
  $$\begin{aligned} E_{14,24,(1,2,4,8)}=-\frac{1}{18432} E_2 \big |\left( 1-V_3\right) \big |S_{24,15}\big |V_4. \end{aligned}$$
  $$\square $$

As a corollary to Proposition 4.1, we obtain explicit lower bounds on the Fourier coefficients $a_{r,M,{\varvec{\alpha }}}(n)$ in these special cases.

### Corollary 4.2

Let $$n\in \mathbb {N}$$.

1. If $n\equiv 15\ \, \left( {\text {mod}} \, 40 \right)$, then we have
  $$\begin{aligned} a_{7,10,(1,2,4,8)}(n)\ge \frac{n}{240}. \end{aligned}$$
2. If $n\equiv 39\ \, \left( {\text {mod}} \, 56 \right)$, then we have
  $$\begin{aligned} a_{9,14,(1,2,4,8)}(n)\ge \frac{n}{672}. \end{aligned}$$
3. If $n\equiv 28\ \, \left( {\text {mod}} \, 64 \right)$, then we have
  $$\begin{aligned} a_{10,16,(1,2,4,8)}(n)\ge \frac{n}{1024}. \end{aligned}$$
4. If $n\equiv 15\ \, \left( {\text {mod}} \, 72 \right)$, then we have
  $$\begin{aligned} a_{11,18,(1,2,4,8)}(n)\ge \frac{n}{1728}. \end{aligned}$$
5. Assume that $80\mid n$ and write $n=2^a5^bc$ with $\gcd (10,c)=1$. We have
  $$\begin{aligned} a_{12,20,(1,2,4,8)}(n)\ge \frac{5^b c}{120}{\left\{ \begin{array}{ll} 2^{a-4}&{}\text {if }4\le a\le 7,\\ 24&{}\text {if }a\ge 8.\end{array}\right. } \end{aligned}$$
6. If $n\equiv 71\ \, \left( {\text {mod}} \, 88 \right)$, then we have
  $$\begin{aligned} a_{13,22,(1,2,4,8)}(n)\ge \frac{n}{2640}. \end{aligned}$$
7. If $n\equiv 60\ \, \left( {\text {mod}} \, 96 \right)$, then we have
  $$\begin{aligned} a_{14,24,(1,2,4,8)}(n)\ge \frac{n}{3072}. \end{aligned}$$

### Proof

For $m\ne 12$, the claims with the exception of (5) follow directly from Proposition 4.1. For (5), a direct simplification yields that the right-hand side of Proposition 4.1 (5) simplifies as

$$\begin{aligned} \frac{5^b\sigma (c)}{120} {\left\{ \begin{array}{ll} 2^{a-4}&{}\text {if }4\le a\le 7,\\ 24&{}\text {if }a\ge 8,\end{array}\right. } \end{aligned}$$

which gives the claim.$\square$

## Proof of Theorem 1.2

In this section, we prove Theorem 1.2. The constants $C_m$ from the theorem statement may be found in Table 2.

### Proof of Theorem 1.2

We require the case $\ell =4$ of Lemma 2.5. Since the inner sum only has a single term namely $m=0$ in this case, Lemma 2.5 simplifies as

5.1 $$\begin{aligned}{} & {} \Vert f_{r,M,{\varvec{\alpha }}}\Vert ^2\le \frac{2\cdot 3^{6}}{\pi ^{4}} \frac{M^{4}N_{{\varvec{\alpha }}}^{2}}{\prod _{p\mid M^2N_{{\varvec{\alpha }}}}\left( 1-p^{-2}\right) }\nonumber \\{} & {} \quad \times \sum _{\delta \mid M^2N_{{\varvec{\alpha }}}}\varphi \left( \frac{M^2N_{{\varvec{\alpha }}}}{\delta }\right) \varphi (\delta )\frac{M^2N_{{\varvec{\alpha }}}}{\delta }\left( \frac{\gcd (M^2,\delta )}{M^2}\right) ^{4} 2 \left( \frac{27}{D_{{\varvec{\alpha }}}} \frac{M^2N_{{\varvec{\alpha }}}}{\pi } +16\right) .\qquad \quad \end{aligned}$$

For $\theta _{r,M,(1,2,4,8)}$, we obtain a lower bound for $a_{r,M,(1,2,4,8)}(n)$ (for *n* in an appropriate congruence class) from Corollary 4.2 (see the third column of Table 1 for a list of the bounds for individual choices of *r* and *M*).

**Table 1:** Bounds for $a_{r,M,(1,2,4,8)}$, $\Vert f_{r,M,(1,2,4,8)}\Vert$, and $|b_{r,M,(1,2,4,8)}|$

*r*	*M*	Bound for $a_{r,M,(1,2,4,8)}$	Bound for $\Vert f_{r,M,(1,2,4,8)}\Vert$	Bound for $|b_{r,M,(1,2,4,8)}|$
7	10	$\frac{n}{240}$	$8.11\cdot 10^{14}$	$3.41\cdot 10^{30} n^{\frac{3}{5}}$
9	14	$\frac{n}{672}$	$1.03\cdot 10^{16}$	$3.48\cdot 10^{31} n^{\frac{3}{5}}$
10	16	$\frac{n}{1024}$	$3.2\cdot 10^{16}$	$9.98\cdot 10^{31} n^{\frac{3}{5}}$
11	18	$\frac{n}{1728}$	$6.1\cdot 10^{16}$	$1.52\cdot 10^{32} n^{\frac{3}{5}}$
12	20	$\frac{n}{1920}$	$1.49\cdot 10^{17}$	$3.69\cdot 10^{32}n^{\frac{3}{5}}$
13	22	$\frac{n}{2640}$	$2.55\cdot 10^{17}$	$6.96\cdot 10^{32}n^{\frac{3}{5}}$
14	24	$\frac{n}{3072}$	$5.63\cdot 10^{17}$	$1.09\cdot 10^{33} n^{\frac{3}{5}}$

Computing the constants in (5.1) explicitly for fixed *M* yields an upper bound for $\Vert f_{r,M,(1,2,4,8)}\Vert ^2$ (see the fourth column of Table 1 for the explicit bounds), which plugged into Lemma 2.4 yields an upper bound for $|b_{r,M,(1,2,4,8)}(n)|$ (see the final column of Table 1 for the explicit bounds). Plugging the bounds for $a_{r,M,(1,2,4,8)}(n)$ and $|b_{r,M,(1,2,4,8)}(n)|$ into (2.1), we see that $s_{r,M,(1,2,4,8)}(n)>0$ for *n* sufficiently large in an appropriate congruence class (see Table 2 for the explicit constants).

**Table 2:** Bounds on *n* for $s_{m,2(m-2),(1,2,4,8)}(n)>0$ and $r_{m,(1,2,4,8)}>0$

*m*	Bound for $s_{m,2(m-2),(1,2,4,8)}(n)>0$	Bound $C_m$ for $r_{m,2(m-2),(1,2,4,8)}(n)>0$
7	$1.92\cdot 10^{82}$	$4.8\cdot 10^{80}$
9	$8.38\cdot 10^{85}$	$1.5\cdot 10^{84}$
10	$3.41\cdot 10^{87}$	$5.33\cdot 10^{85}$
11	$3.55\cdot 10^{88}$	$4.93\cdot 10^{86}$
12	$4.25\cdot 10^{89}$	$5.31\cdot 10^{87}$
13	$4.57\cdot 10^{90}$	$5.19\cdot 10^{88}$
14	$2.04\cdot 10^{91}$	$2.13\cdot 10^{89}$

We then conclude that $r_{m,(1,2,4,8)}>0$ for *n* sufficiently large by using (1.3), yielding the claim. $\square$

In order to explain why it is sufficient to assume that $n\not \equiv 4\ \, \left( {\text {mod}} \, 16 \right)$ for $m=12$ in Theorem 1.2, we require the following lemma combined with (1.3).

### Lemma 5.1

Let $n\in \mathbb {N}$ be given. If the equation

$$\begin{aligned} x_1^2+2x_2^2+4x_3^2+8x_4^2=n \end{aligned}$$

is solvable with $x_j\equiv 12\ \, \left( {\text {mod}} \, 20 \right)$, then the equation

$$\begin{aligned} x_1^2+2x_2^2+4x_3^2+8x_4^2=256n \end{aligned}$$

is also solvable with $x_j\equiv 12\ \, \left( {\text {mod}} \, 20 \right)$.

## Footnotes

1 We denote vectors like ${\varvec{\alpha }}$ in bold and the *j*-th component of a vector ${\varvec{\alpha }}$ we write as $\alpha _j$ throughout.
